# A Curious Case of Proximal Muscle Weakness with Eosinophilic Polymyositis

**DOI:** 10.1155/2016/7810916

**Published:** 2016-09-14

**Authors:** Ciel Harris, Robert Ali, Julio Perez-Downes, Firas Baidoun, Marianne DeLima, Jaimin Shah, Win Aung, Raafat F. Makary

**Affiliations:** ^1^Internal Medicine Department, University of Florida College of Medicine, Jacksonville, FL, USA; ^2^Neurology Department, University of Florida College of Medicine, Jacksonville, FL, USA; ^3^Pathology and Laboratory Medicine Department, University of Florida College of Medicine, Jacksonville, FL, USA

## Abstract

Eosinophilic polymyositis (EPM) is part of a rare disorder, eosinophilic myopathies (EM), which is a form of polymyositis characterized by the presence of eosinophils in muscle biopsy sections and occasionally blood eosinophilia. Herein, we are presenting an interesting case of eosinophilic polymyositis presenting with muscle pain with no other organ systems involved.

## 1. Introduction

Eosinophilic myopathies (EM) comprise a clinically and pathologically heterogeneous group of rare diseases that include three main subtypes: focal eosinophilic myositis, eosinophilic polymyositis, and eosinophilic perimyositis. We present a case of eosinophilic polymyositis, a rare disease with few cases reported in the literature [1, 2].

## 2. Case Report

A 61-year-old Caucasian male presented with progressive muscle weakness for two months. He initially noted difficulty with standing from a seated position, which progressed to weakness with ambulation. One week prior to presentation, he experienced difficulty brushing his hair and lifting objects above his head. He denied constitutional symptoms. He has a history of chronic alcohol abuse, averaging four beers/day for the past few years. He also endorsed a 50-pack-year smoking history. There is no family history of muscle disease. On examination he demonstrated 4/5 proximal muscle weakness at both upper and lower extremities, with an otherwise unremarkable neurologic examination. There were no dermatologic manifestations.

**Figure 1:**
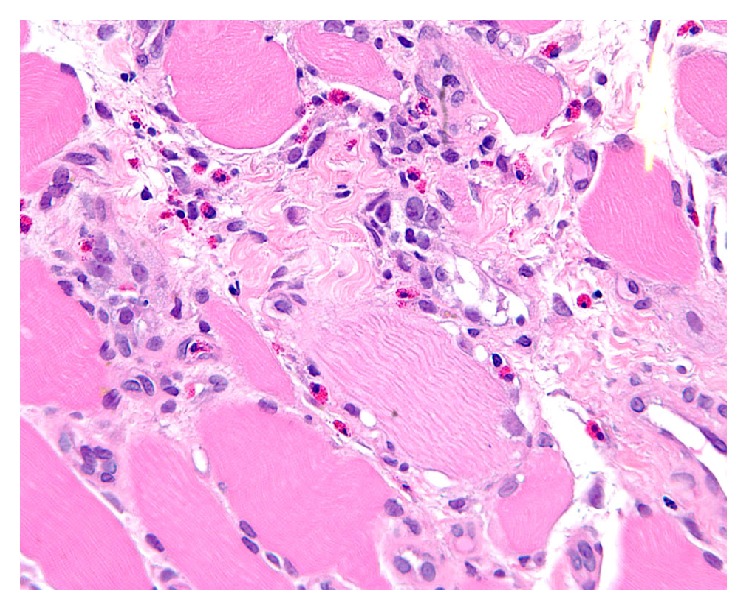
Biopsy of left vastus lateralis showing an abundance of eosinophils.

Creatine kinase (CK) was increased at 8131 U/L, with concomitant elevated inflammatory markers, erythrocyte sedimentation rate (ESR) of 35 mm/hr, and C-reactive protein of 121.7 mg/L. A complete blood count was significant for an eosinophilia of 17.4% with absolute eosinophils count of 940/mm^3^ (0.94 × 10^9^/L). Serologies for* T. gondii* and* T. solium* were nonreactive. A CT of the abdomen, chest, and pelvis showed no evidence of malignancy. A myositis antibody panel, which included serologies for Mi-2, PM-SCL, PL-7, PL-12, EJ, OJ, KU, SRP, and U2 SN RNP, was negative. Electromyography demonstrated myopathic motor unit potentials, fibrillation potentials, and positive sharp waves, consistent with an “irritable” myopathy. Core muscle biopsy of the vastus lateralis (Figure 1) demonstrated several foci of perimysial and endomysial infiltrate consisting primarily of lymphocytes and eosinophils. During his initial hospitalization, he had spontaneous normalization of his muscle strength and CK over five days. He was discharged without immunosuppressive therapy and was seen in follow-up two months later and continued to be in remission.

## 3. Discussion

EM is a rare group of heterogeneous conditions that are characterized by the presence of peripheral and/or muscle eosinophilia. There are three major subsets of EM: focal eosinophilic myositis, eosinophilic polymyositis, and eosinophilic perimyositis [1, 2]. The latter is typically associated with eosinophilic inflammatory infiltrates in the superficial and deep fascia in addition to muscle. In our patient, inflammatory infiltrates were confined primarily in the endomysium and perimysium of muscle, which is not consistent with perimyositis. These characteristics in tandem with the patient's clinical presentation of generalized proximal muscle weakness satisfy previously proposed diagnostic criteria of polymyositis [3].

There are several systemic causes of eosinophilic infiltration of the muscle, including parasitic infection, Churg-Straus granulomatosis, or various drugs or toxins. EM can also be associated with both hematologic and solid tumor malignancies. It can also occur as a component of idiopathic hypereosinophilic syndrome (HES) [4, 5]; however our patient does not satisfy the accepted diagnostic criteria for HES [6]. Generally, the prognosis of idiopathic EM is good, especially in the localized forms of focal EM. Eosinophilic polymyositis and perimyositis can benefit from glucocorticoid therapy.

Although inflammatory myopathies occasionally have a relapsing and remitting course, spontaneous remission without immunotherapy is rare. Remission without immunotherapy in patients with eosinophilic myositis has not been previously reported. Long-term follow-up of this patient is required to observe for relapses.

## 4. Conclusion

Idiopathic eosinophilic polymyositis is a rare form of inflammatory myopathy. Diagnosis evaluation to exclude systemic causes of eosinophilia is required in these patients [1, 2]. The prognosis of idiopathic eosinophilic polymyositis is good. Our case presentation describes a patient with idiopathic eosinophilic polymyositis who achieved spontaneous remission and contributes to the few reported cases in the literature. Long-term follow-up is needed to determine if remission will be sustained.
